# Dissection of Protein Interactomics Highlights MicroRNA Synergy

**DOI:** 10.1371/journal.pone.0063342

**Published:** 2013-05-14

**Authors:** Wenliang Zhu, Yilei Zhao, Yingqi Xu, Yong Sun, Zhe Wang, Wei Yuan, Zhimin Du

**Affiliations:** Institute of Clinical Pharmacology, The Second Affiliated Hospital of Harbin Medical University, Harbin, China; University of Westminster, United Kingdom

## Abstract

Despite a large amount of microRNAs (miRNAs) have been validated to play crucial roles in human biology and disease, there is little systematic insight into the nature and scale of the potential synergistic interactions executed by miRNAs themselves. Here we established an integrated parameter synergy score to determine miRNA synergy, by combining the two mechanisms for miRNA-miRNA interactions, miRNA-mediated gene co-regulation and functional association between target gene products, into one single parameter. Receiver operating characteristic (ROC) analysis indicated that synergy score accurately identified the gene ontology-defined miRNA synergy (AUC = 0.9415, *p*<0.001). Only a very small portion of the random miRNA-miRNA combinations generated potent synergy, implying poor expectancy of widespread synergy. However, targeting more key genes made two miRNAs more likely to act synergistically. Compared to other miRNAs, miR-21 was a highly exceptional case due to frequent appearance in the top synergistic miRNA pairs. This result highlighted its essential role in coordinating or strengthening physiological and pathological functions of other miRNAs. The synergistic effect of miR-21 and miR-1 were functionally validated for their significant influences on myocardial apoptosis, cardiac hypertrophy and fibrosis. The novel approach established in this study enables easy and effective identification of condition-restricted potent miRNA synergy simply by concentrating the available protein interactomics and miRNA-target interaction data into a single parameter synergy score. Our results may be important for understanding synergistic gene regulation by miRNAs and may have significant implications for miRNA combination therapy of cardiovascular disease.

## Introduction

MicroRNAs (miRNAs) can be classified as one super-family of small single-stranded non-coding RNA molecules that play roles in posttranscriptional gene regulation for fine-tuning protein abundance [1]. Up to now, there have been more than 2000 known mature human miRNA transcript records that can be retrieved from the 19.0 release of miRBase [2]. As the selective gene targeting of miRNAs is widely explored, the veil of mystery is being revealed about the mechanisms underlying their pervasive roles in human biology and disease [3]. For example, there has been 276 literature records of human target genes of miR-21, one of the most concerned miRNAs by far [4], [5], in the miRNA-target interaction database miRSel that updates daily [6]. Thousands of miRNAs constitute into an additional functional layer in our biological system and contribute to its complexity, which has been recognized only until recently. By means of network-based information integration of the currently available mass data of miRNA-target interactions and functional protein association, we revealed that miR-21 might play a core role in biological functions of major human tissues due to its preferential binding to ubiquitously expressed mRNAs [7].

Despite that our knowledge about individual nodes of the miRNA network are rapidly accumulating, little is known about functional association and interactions between them. Recently, Xu *et al.* suggested that miRNAs can impose synergistic gene regulation and demonstrate functional connections owing to targeting of common genes, collaboratively participating in the same processes [8]. The miRNA-miRNA interaction network implies the existence of extensive synergies in the miRNA world, in which slight expression disturbance of miRNAs could collaboratively generate profound biological effects. However, whether the synergistic actions could happen between miRNAs that target different genes was unknown. We proposed that these two mechanisms can jointly contribute to synergistic miRNA actions. To test this hypothesis, we developed an integrated parameter synergy score which can be used to assess the nature and scale of miRNA synergy in human genome. This unique scoring system takes both the incorporative contributions of miRNA co-regulation and the intrinsic functional links between target gene products into account as mechanisms for miRNA synergy.

As the functional roles of miRNAs are continually brought to light in cardiovascular biology and disease, main concerns have been prospectively and broadly discussed on the potential application of miRNAs in clinical settings [9], [10]. Comprehensive understanding of the operation principles in the new biological regulation layer is definitely needed [11]–[13], despite it is widely agreed that restoration or reconstruction of their served roles in posttranscriptional gene regulation may bring benefits to the injured heart. Systematic exploration into miRNA synergy could undoubtedly deepen the awareness of collaborative relationship between miRNAs. The synergy score calculation method established here enables easy and accurate identification of potent miRNA synergy under the only premise that miRNA-target interaction and protein interactomics data are available to given gene sets of common characteristics, such as apoptosis-related genes [14] or cardiac-expressed genes [15]. Our findings may offer a theoretical framework and guideline for combinational miRNA therapy of cardiovascular disease, for instance, myocardial ischemia (MI) and heart failure (HF).

## Results

### Definition and Validation of Synergy Score

**Figure 1:**
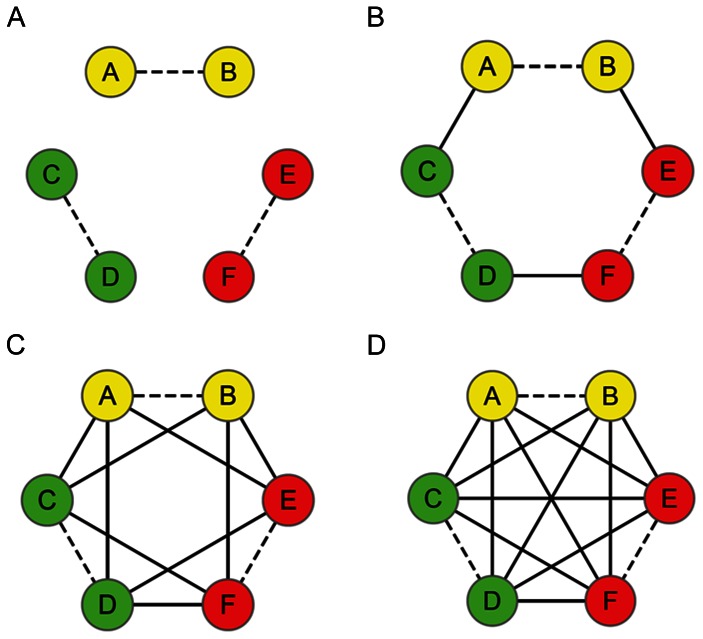
Illustration of PIS calculation. Suppose miRNA *i* targets genes a, b, c and d that encode protein A, B, C and D, respectively; miRNA *j* targets genes a, b, e and f that encode protein A, B, E and F, respectively. The six proteins can be divided into three clusters according to diverse miRNA regulations, that is, protein A and B (yellow nodes, regulated by both miRNA a and b); protein C and D (green nodes, regulated by miRNA a only); protein E and F (red nodes, regulated by miRNA b only). Four possible conditions of functional association between protein clusters were compared by calculating PIS. PIS is equal to the sum of proteins (n = 6) divided by the sum of protein-protein interactions (PPIs) between protein clusters. A. No functional association between protein clusters (0 PPI, PIS = 0); B. Weak functional association between protein clusters (3 PPIs, PIS = 0.50); C. Moderate functional association between protein clusters (9 PPIs, PIS = 1.50); D. The highest level of functional association between protein clusters (12 PPIs, PIS = 2.00). The dash and solid edges represent PPIs inside and between protein clusters, respectively. PIS: protein interaction score.

MiRNA synergy score has been applied to determining whether two given miRNAs tend to act synergistically under physiological and pathological conditions. It comprises two independent parameters, the target similarity score (TSS) and the protein interaction score (PIS). TSS and PIS quantitatively measure the degree of gene co-regulation by two miRNAs and functional association between their target gene products, respectively. Because neither TSS nor PIS alone is sufficient to explain miRNA synergy, we integrated them into a new parameter, designated as integrated parameter synergy score (see **Materials and Methods**). The TSS calculation is based upon the reliable miRNA-target interaction data; we therefore employed two related databases miRSel and ExprTargetDB [16] in combination. For PIS computation, the experimentally validated protein interactomics data was also acquired by using the Cytoscape [17] plugin BisoGenet [18]. Figure 1 is the graphic illustration of the methodology of PIS acquisition with two miRNAs *i* and *j* as an example. The TIS score of miRNAs *i* and *j* was constant in the four scenes with different numbers of functional protein-protein interactions (PPIs). PPIs are an indication that the involving proteins tend to form a functional module and jointly participate in the same biological process. Simply put, even the miRNAs targeting different genes could elicit synergistic effects. As shown in Figure 1, PIS values can be used to measure the increase of the number of PPIs between protein clusters that are under different miRNA regulation.

By calculating PIS, we can easily evaluate how close the association is between the protein products encoded by the target genes of different miRNAs (Figure S1 and Table S1). Due to the widespread lack of necessary functional association between proteins encoded by target genes, no expectance could be made that random miRNA-miRNA combinations would generally participate in a higher number of co-regulated biological processes (Figure S2). On the contrary, closer functional protein association might lead to frequent involvement of two irrelevant miRNAs into the same biological processes. For example, miR-1 and miR-21 are located at human chromosome 20 and 17, respectively. Low TSS value of merely 0.067 indicated disparate binding selectivity of these two miRNAs to mRNAs; however, high PIS value of 1.542 strongly suggested collaborative participation in the same processes and potential synergy between them. Consistently, gene ontology (GO) analysis revealed that miR-1 and miR-21 were simultaneously implicated in more than 30 GO processes. PIS instead of TSS was positively correlated with the number of miRNA co-regulated GO-term processes, but synergy score gave the best indication for potential miRNA co-regulated GO-term processes (Figure S2). Specifically, as synergy score was equal to or more than two, extremely high number of miRNA co-regulated GO-term processes could be confidently expected, strongly suggesting miRNA synergy.

It is known that each single miRNA has the potential to regulate multiple target genes, from decades to hundreds. Thus far, more than 2000 mature miRNAs have been identified from the human genome. To obtain true and unique synergy scores of miRNA pairs, thorough understanding of miRNA targets is a prerequisite. Unfortunately, however, our present knowledge about miRNA targets is still rather incomplete. In the pilot examination of our new strategy for miRNA synergy prediction, we found that a threshold of 50 targets per miRNA yielded reasonably reliable and accurate calculation of synergy scores (Figure S3). Based on this initial analysis, we set a criterion that only the miRNAs that have been experimentally verified or are theoretically predicted to regulate expression of a minimum number of 50 protein-encoding genes [19] were recruited for our assessment of their potential synergistic interactions. In this way, we were able to include only 99 out of >2000 human miRNAs in our experimental validation (Table S2). In a random manner, there were totally 4851 (99×98/2) pair-wise miRNA combinations for our validation.

To expand the range of application of our method, we repeated the same analyses based on another target gene prediction database miRecords (http://mirecords.umn.edu/miRecords/) to retrieve reliable miRNA target genes, which integrates at least five target prediction algorithms [20]. By replacing ExprTargetDB with miRecords, the number of miRNAs that met our restricted standard for the number of target genes included for assessing the potential synergy were increased from 99 to 293 (Table S3). Not surprisingly, qualitatively the same results were obtained, indicating the robustness of our method (Table S4). Subsequently, we also examined the robustness of our method against PPI networks. After merging the manually curated PPIs in the Human Signaling Network dataset that is updated just recently with the experimentally validated PPIs [18], [21], we re-calculated the synergy scores for the 99 miRNAs (Table S2). Not surprisingly, despite that different network data was used, potent synergistic miRNA pairs could be still highlighted among the 4851 randomly pair-wise miRNA combinations (Table S5).

**Figure 2:**
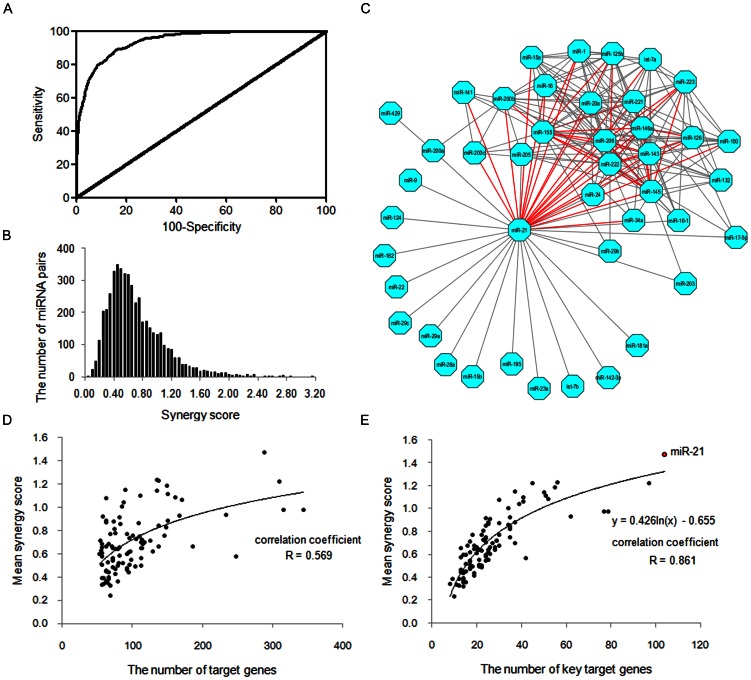
Results of miRNA synergy scores in the whole human genome. A. receiver operating characteristic (ROC) curve of synergy score for distinguishing between the high and low co-regulation groups of miRNA pairs; The area under the ROC curve of synergy score is 0.9415 (*p*<0.0001). B. Frequency distribution of miRNA synergy scores; C. Potential synergistic miRNA pairs in the whole human genome (synergy score ≥1.500). Red edge represents potent miRNA synergy in all of the 4851 miRNA pairs analyzed here (synergy score ≥2.000). D. Semi-log correlation between the number of miRNA target genes and the mean miRNA synergy score (correlation coefficient R = 0.569); E. Semi-log correlation between the number of key miRNA target genes and the mean miRNA synergy score (correlation coefficient R = 0.861); Superscript a: the key miRNA target genes are defined here as genes that encode hub proteins of high betweenness centrality in the global protein interaction network (degree ≥20 and betweenness centrality >0.001). MiR-21 is highlighted as red dot.

Joint participation in the same processes implies potential miRNA synergy. In our work, receiver operating characteristic (ROC) analysis validated that synergy score is able to effectively pick up synergistic miRNA pairs from the random miRNA-miRNA combinations (Figure 2A) with the correction factor α being optimized and set at 1.8 (Figure S4). No significant dependences of α and synergy score's performance were found on the cutoff of the number of co-regulated GO terms in the ROC analysis (Figure S5). In comparison with the areas under the ROC curves of TSS and PIS (0.8384 and 0.9397, respectively), the value of 0.9415 for the integrated parameter synergy score turned out to be the highest one. This result indicates that the occurrence of synergistic miRNA actions was due to the consolidated outcome of miRNA binding to common genes and functional association between gene products. Synergy score possessed the best performance for predicting potential miRNA synergy. Furthermore, the greater value of the area under curve (AUC) manifested that synergy score can give accurate prediction of miRNA synergy. More importantly, only miRNA-target interaction and PPI data are required for synergy score calculation, which greatly simplifies the analysis. Figure 2B shows the frequency distribution of synergy score values for 4851 random miRNA-miRNA pair combinations. In general, the synergy score values of most of the miRNA pairs were low. This implies that only a very small proportion of potent synergistic miRNA pairs would be expected in the whole miRNA regulation layer, instead of extensive miRNA synergy suggested by previous work, in which only the gene co-regulation was considered to cause miRNA synergy [8].

Surprisingly, we noted that miR-21 frequently appeared as the top miRNA synergistic pairs (Figure 2C). By dissecting protein connectivity, subsequent network topology analysis indicated that prominent tendency in binding to a large number of key genes conferred miR-21 the vital roles in global miRNA synergy (Figure 2D and E). Here, key genes refer specifically to the genes that encode hub proteins of high betweenness centrality (see **Materials and Methods**). High degree and betweenness centrality represent multiple important protein functions [22]. The number of miR-21 target genes is not the largest among the 99 miRNAs analyzed (Table S2), but it was predicted to be involved into the highest amount of significantly over-represented biological processes as GO analysis reveals (GO term, n = 182). A random assignation test was then designed to ascertain whether targeting a higher proportion of key genes is the reason for miRNAs to participate in more GO processes (Figure S6). Our result strongly suggested that targeting key genes make miRNA regulation appear to be more pervasive even in the assumed random modeling. Intriguingly, even an averaged GO process sum of 53.9 could be expected for the fictitious miRNA in our test even though all the target genes were randomly selected as key genes. This in turn emphasized the extremely high selectivity of the real miRNAs on posttranscriptional gene regulation.

### Cardiac miRNA Synergy

**Figure 3:**
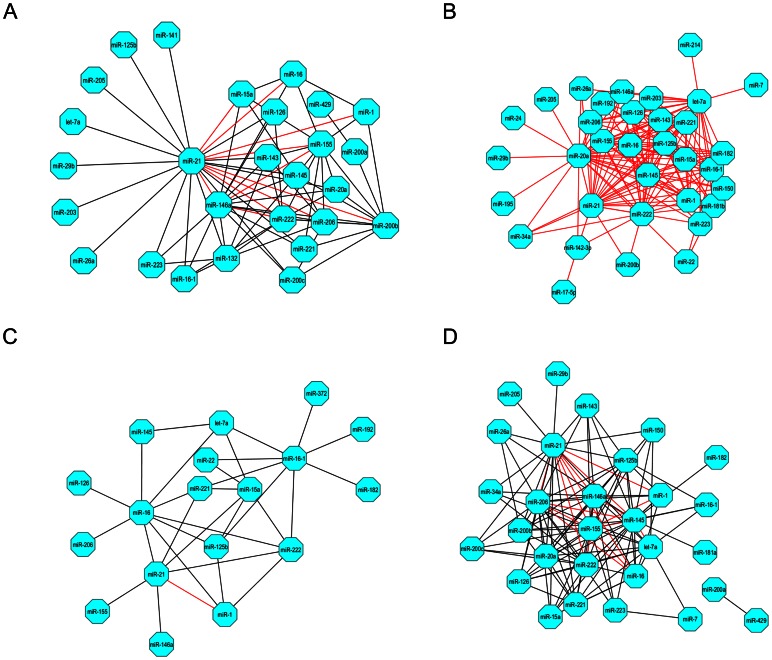
Results of miRNA synergy scores in restricted conditions. A. The cardiac miRNA synergistic network (synergy score ≥1.500); B. The miRNA synergistic network on apoptosis-related genes (synergy score ≥2.000); C. The miRNA synergistic network on MI-associated genes (synergy score ≥1.500); D. The miRNA synergistic network on HF-associated genes (synergy score ≥1.500). Notably, in each network red edge represents potent miRNA synergy in all of the 4851 miRNA pairs analyzed here (synergy score ≥2.000).

We investigated miRNA synergy under tissue-restricted condition. Only cardiac protein interactomics data were applied to the functional association between target gene products and calculate miRNA synergy scores (Table S6). As expected, miR-21 was located at the hub of cardiac miRNA synergy network due to its ability to regulate a large quantity of cardiac key genes (Figure 3A and S7). Although only half of the global miRNA-target interactions were found to actually occur in the heart tissue, the majority of potential synergistic miRNA pairs were preserved as indicated by the result of synergy score calculation (Figure S8). Taken together with our current knowledge about the tissue-specific miRNA regulation [7], our result further suggested that a relatively stable framework of synergistic miRNA regulation system may ubiquitously exist in human tissues and organs for the maintenance and timely adjustment of the essential cellular activities. In a functional study of cardiac progenitor cell transplantation, Hu *et al.* [23] found that transduction with a miRNA cocktail (miR-21, miR-24 and miR-221) generated the highest cell viability of cardiac progenitor cell, implying synergy between miRNAs. This result is consistent with our findings. MiR-21 and miR-221 co-regulate 56 GO processes and were arranged in a synergistic miRNA pair. Extremely high synergy score values were calculated, 2.607 in whole genome and 2.049 for cardiac genes. Their synergistic effects on the morphology and function of cardiac myocytes are still unknown.

### MiRNA Synergistic Regulation on Apoptosis

**Figure 4:**
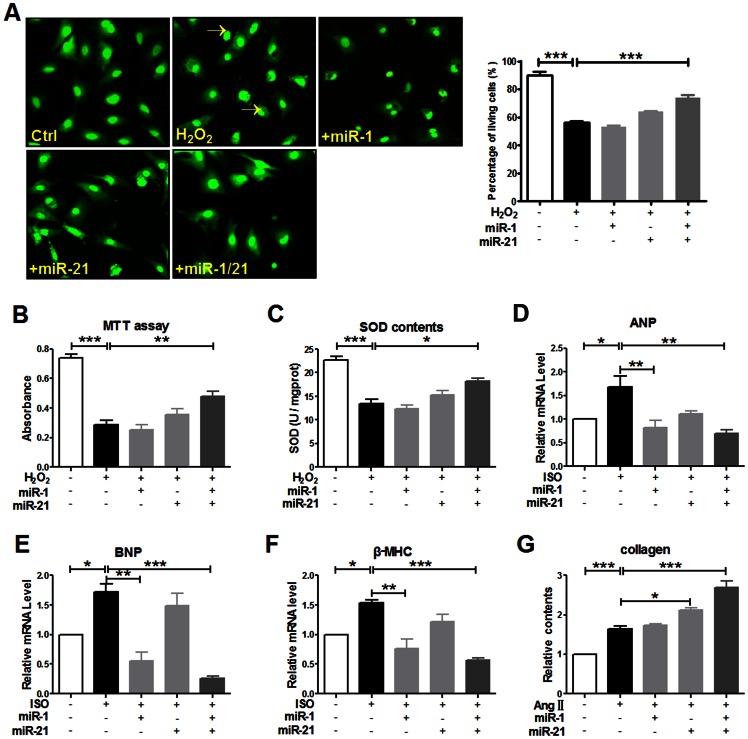
Experimental results of synergistic actions of miR-1 and miR-21. A. Results of AO/EB staining (neonatal rat ventricular CMs, 100×magnification). The yellow arrows point to the apoptotic cells. Left panel: result of cell counting. ***p<0.001 Ctrl *vs* H_2_O_2_ treatment group; ***p<0.001 miR-1 and miR-21 co-transfection group *vs* H_2_O_2_ treatment group; At least a sum of 100 cells were counted in four fields per group. B. Results of MTT assay (neonatal rat ventricular CMs). **p<0.01, ***p<0.001 *vs* H_2_O_2_ treatment group; n = 5. C. Measurement of SOD contents (neonatal rat ventricular CMs). *p<0.05, ***p<0.001 *vs* H_2_O_2_ treatment group; n = 5. D. Quantitative RT-PCR results of gene expression of ANP (neonatal rat ventricular CMs). *p<0.05, **p<0.01 *vs* ISO treatment group; n = 5. E. Quantitative RT-PCR results of gene expression of BNP (neonatal rat ventricular CMs). *p<0.05, **p<0.01, ***p<0.001 *vs* ISO treatment group; n = 5. F. Quantitative RT-PCR results of gene expression of β-MHC (neonatal rat ventricular CMs). *p<0.05, **p<0.01, ***p<0.001 *vs* ISO treatment group; n = 5. G. Measurement of collagen contents (neonatal rat CFs). *p<0.05, ***p<0.001 *vs* AngII treatment group; n = 5. AO/EB staining: Acridine orange/ethidium bromide staining; CMs: cardiomyocytes; CFs: cardiac fibroblasts; quantitative RT-PCR: quantitative reverse transcription–polymerase chain reaction. ANP: atrial natriuretic peptide; BNP: brain natriuretic peptide; β-MHC: beta myosin heavy chain; ISO: isoproterenol; AngII: angiotensinII.

**Figure 5:**
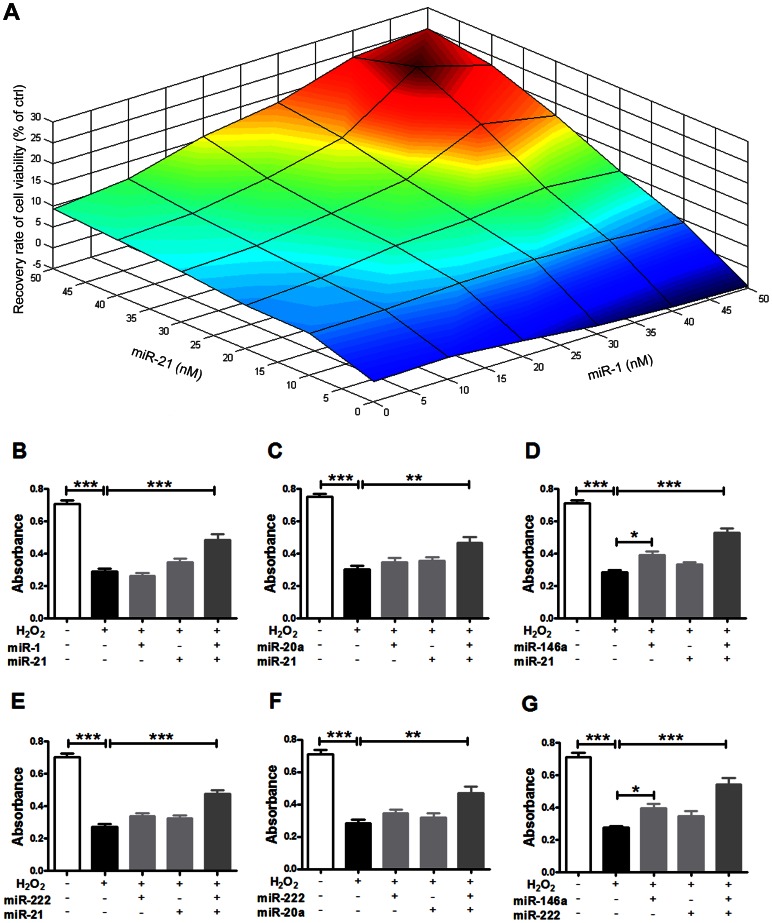
Synergisitic anti-apoptotic properties of miRNA pairs against H_2_O_2_-induced myocardial cell injury. A. Heatmap of recovery rate of cell viability at different transfection concentration ratios of miR-1 and miR-21. Recovery rate of cell viability is calculated as the difference between the mean absorbance values of miRNA transfection group and H_2_O_2_ treatment group divided by that of control group (expressed in percentage); n = 5. B. Results of MTT assay of the miRNA pair miR-1:miR-21 (40 nM). ***p<0.001 ctrl *vs* H_2_O_2_ treatment group; ***p<0.001 miR-1 and miR-21 co-transfection group *vs* H_2_O_2_ treatment group; n = 5. C. Results of MTT assay of the miRNA pair miR-20a:miR-21 (40 nM). **p<0.01, ***p<0.001 vs H_2_O_2_ treatment group; n = 5. D. Results of MTT assay of the miRNA pair miR-21:miR-146a. *p<0.05, miR-146a transfection group *vs* H_2_O_2_ treatment group; ***p<0.001 ctrl *vs* H_2_O_2_ treatment group; ***p<0.001 miR-21 and miR-146a co-transfection group *vs* H_2_O_2_ treatment group; n = 5. E. Results of MTT assay of the miRNA pair miR-21:miR-222 (40 nM). ***p<0.001 ctrl *vs* H_2_O_2_ treatment group; ***p<0.001 miR-21 and miR-222 co-transfection group *vs* H_2_O_2_ treatment group; n = 5. F. Results of MTT assay of the miRNA pair miR-20a:miR-222 (40 nM). **p<0.01, ***p<0.001 vs H_2_O_2_ treatment group; n = 5. G. Results of MTT assay of the miRNA pair miR-146a:miR-222. *p<0.05, miR-146a transfection group *vs* H_2_O_2_ treatment group; ***p<0.001 ctrl *vs* H_2_O_2_ treatment group; ***p<0.001 miR-146a and miR-222 co-transfection group *vs* H_2_O_2_ treatment group; n = 5.

Increasing lines of evidence have well demonstrated that dysregulation of mechanisms controlling apoptosis (programmed cell death) plays a central role in human pathology [24]. In this study, we also explored miRNA-mediated synergistic regulation on apoptosis-related genes by computing miRNA synergy scores (Table S7). Compared to the moderate miRNA regulation densities upon the genome-wide and cardiac-expressed genes, apoptosis-related genes were under a more intensive control exerted by miRNAs (Table S8). We constructed the synergistic miRNA network on cell apoptosis (Figure 3B). The multi-faceted miRNA miR-21 still stood as an irreplaceable hub in the network [25], [26]. Notably, miR-1 was found to act synergistically with miR-21 on cell apoptosis as synergy score confidently predicted. The following biological experiments further supported this notion (Figure 4A and B). At a low transfection concentration of 50 nM, neither miR-1 nor miR-21 significantly altered H_2_O_2_-induced apoptosis of cultured neonatal rat cardiomyocytes as our MTT assay results indicated (Figure 4 and S9). However, co-transfection of miR-1 and miR-21 elicited a remarkable anti-apoptosis effect, strongly suggesting a potent synergy (*p*<0.01). Furthermore, we investigated the optimal transfection concentration ratio of miR-1 and miR-21 and found that the maximum anti-apoptosis effect was achieved as they were co-transfected at 40 nM (Figure 5A and B). Apart from the miRNA pair miR-1 and miR-21, more miRNA pairs showed synergistic anti-apoptosis against H_2_O_2_-induced myocardial cell injury when co-transfected at 40 nM. This result was consistent with synergy score calculation (Figure 5C–G, Table S7).

### MiRNA Synergy in Cardiovascular Disease

**Figure 6:**
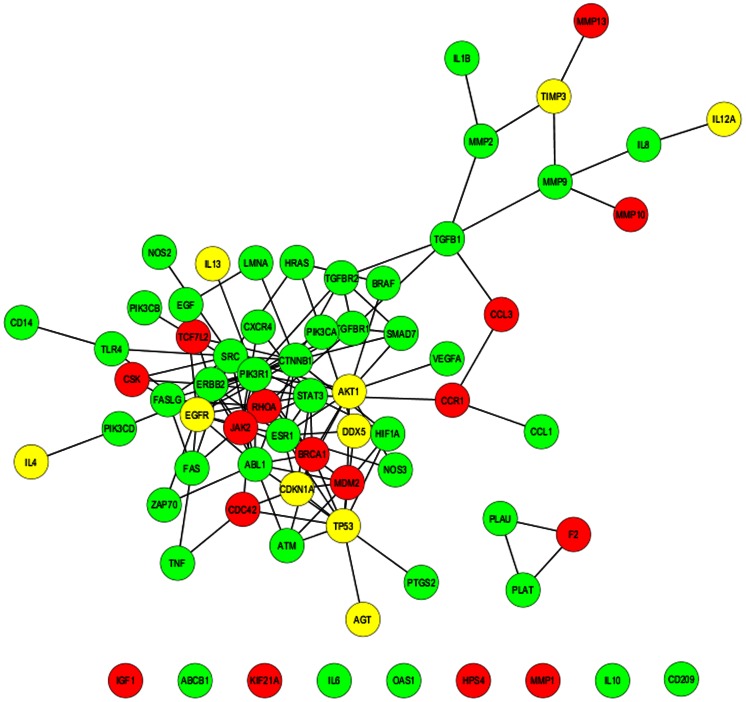
Fibrosis-related network of functional protein association that is regulated by miR-1 and miR-21. Red, green and yellow nodes represent proteins that are encoded by gene targeted by miR-1, miR-21 and both of them, respectively. TSS_miR-1:miR-21_ = 0.169; PIS_miR-:miR-21_ = 1.029; synergy score_miR-1:miR-21_ = 2.021.

**Figure 7:**
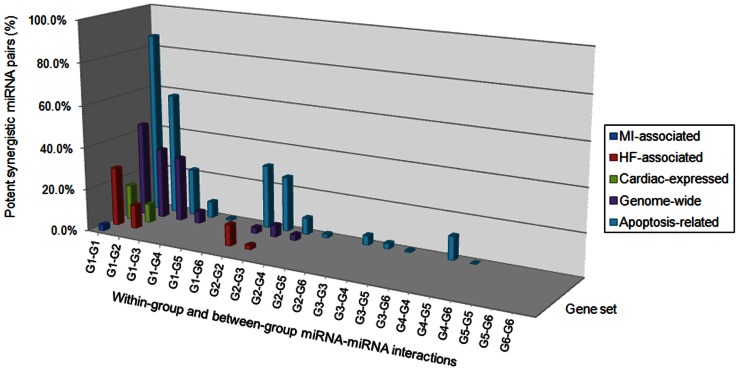
Distribution of potent synergistic miRNA pairs. MiRNA groups according to the number of key target genes: G1 (n >50), G2 (40< n ≤50), G3 (30< n ≤40), G4 (20< n ≤30), G5 (10< n ≤20), G6 (n ≤10).

Recently, numerous studies have confirmed that a large quantity of miRNAs is implicated in cardiovascular disease [27]. And miRNA synergy and its role in cardiac pathology have also attracted interest of researchers. Based on the synergy score calculation results (Table S9 and S10), two synergistic miRNA networks were established for MI and HF associated genes [14], respectively (Figure 3C and D). Relatively high regulation densities on MI- and HF-associated genes by miRNAs revealed biological importance of the two synergistic miRNA networks (Table S8). Many MI- and HF-associated genes are also apoptosis-related (Figure S10). The miR-1:miR-21 miRNA pair was assigned with the highest synergy score among the 4851 random miRNA-miRNA combinations for MI, implying potent synergy (Table S9). Results from our *in vivo* experiments validated that co-transfection of miR-1 and miR-21 significantly ameliorated H_2_O_2_-induced myocardial apoptosis and oxidative stress (Figure 4A–C). In addition, co-transfection of miR-1 and miR-21 also significantly reduced isoproterenol-induced gene expression of ANP, BNP and β-MHC (Figure 4D–F), but obvious synergy was only displayed on BNP, a heart failure biomarker [28]. This result again confirmed our prediction about miRNA synergy using synergy score alone. A very high synergy score value of 2.153 was calculated for the miRNA pair miR-1:miR-21, when only HF-associated target genes were incorporated into computation. As miR-21 is generally recognized for its pivotal role in cardiac fibrosis [29], we investigated whether the potent synergy of miR-1 and miR-21 also operated in fibroblasts. We found that despite that transfection of miR-1 alone failed to affect the collagen content in angiotensin II-treated fibroblasts, significant synergy was still detected when miR-21 was co-transfected with miR-1 (Figure 4G). With fibrosis-related genes being retrieved from the Gene Prospector online tool [14], a high synergy score of miR-1 and miR-21 was obtained which strongly indicates a potential synergistic action of these two miRNAs in cardiac fibrosis (Figure 6). This result was consistent with the significantly promoted fibrosis through miR-1 and miR-21 co-transfection (Figure 4G). Furthermore, we explored the distribution of potent synergistic miRNA pairs in various restricted conditions (synergy score ≥2.0). Interestingly, we found that targeting more key genes would make two miRNAs more likely to act synergistically (Figure 7). This may explain broad synergistic actions afforded by miR-1 and miR-21 (Figure 4).

## Discussion

Acting on hundreds of mRNAs allows the power of individual miRNAs to modulate complex physiological or disease phenotypes. In a recent review about pervasive roles of miRNAs in cardiovascular system, the authors suggested that miRNAs might be more appropriate as rational drug targets, compared to other biological factors [30]. A growing body of evidence has strongly suggested that the novel miRNA-based therapy represents an imperative and promising trend in the future [10]. Despite that successful reversal of pathological processes could be obtained by solely modulating some key miRNAs, we and others [8] suggested that the intrinsic synergy in miRNA regulation may yield more desirable effects because of reduced doses of each miRNA and improved therapeutic selectivity [31]. For this purpose, we performed a network-based assessment on human miRNAs with the hypothesis that miRNA synergy could be revealed by dissecting global protein interactomics.

By integrating the two determinants, TSS and PIS, of miRNA synergy into a single parameter synergy score (see **Materials and Methods**), we successfully identified synergistic miRNA pairs in cardiovascular biology and disease (Figure 2 and 3). Despite that targeting common genes could lead to synergistic action, we found that it was not the main reason for miRNA synergy in comparison with functional protein association that contributed more. In human biology, the proteins with close interactions may comprise of one functional module in the same process. On the contrary, distant proteins in the biological network are likely responsible for different cellular functions. According to the results observed in chemical synergy in cellular signaling pathway [32], we analogized that targeting genes of the same functional module by miRNAs would result in synergistic action regardless of regulatory selectivity of miRNAs–targeting on common genes or different genes. For example, miRNAs miR-1 and miR-21 co-regulate only a small number of genes (TSS = 0.067); however, they jointly participate in more than 30 GO terms in biological processes. Intensive functional protein association can explain this better (PIS_miR-1:miR-21_ = 1.542).

Among the 99 miRNAs analyzed in our study, miR-21 represents one of the most studied miRNAs ever since functional roles of miRNAs have been investigated in human biology and disease [4], [5]. Possible diagnostic and therapeutic applications of this multi-faceted miRNA have been discussed especially in the cardiovascular biology field [26]. This is not just owing to its ubiquitous expression in human tissues [33] but more importantly due to its effects on a large number of key genes that encode hub proteins of high degree and betweenness centrality (Figure 2E and Table S2). Especially for miR-21, it was found to target the greatest number of hub-encoding genes among the thus-far-studied miRNAs (Figure 2E and Table S2). Degree counts the number of neighbors connected to a node. Highly connected nodes defined as the hubs will definitely play more important roles in cellular functions than other proteins [34]. It is very likely that a protein with high betweenness centrality also participates in important cellular functions due to extensive control over PPIs in biological network [35]. Although miRNA-gene interactions can provide us with rich information about the exact roles of miR-21 [6], [16], potential miRNA-miRNA association should not be neglected for better understanding of this magic miRNA in human biology and disease. By calculating synergy score that condensed mega data of miRNA-target interaction and protein interactomics (Table S16), we have a good opportunity to quantitatively assess the status of miR-21 over synergistic miRNA networks (Figure 2C and 3). Interestingly but not surprisingly, the superiority in selective posttranscriptional gene regulation places miR-21 situate in the center of the synergistic miRNA networks within some functionally most important organs such as heart, brain, kidney, liver and lung (Table S6 and Tables S12, S13, S14, S15). This underlines the leading roles of miR-21 in coordinating the functions of other miRNAs in human biology and disease. Our *in vitro* experiment confirmed this finding (Figure 5). Additionally, the synergistic anti-apoptotic effects afforded by miR-21 and miR-221 unraveled in the present study are in line with the synergistic effects of miR-21 and miR-221 in prosurvival of cardiac progenitor cells [23]. Nevertheless, future functional experiments are definitely needed for validating the potential synergy of these miRNAs in the function of cardiac myocytes both in healthy and diseased conditions.

Another important synergistic miRNA-miRNA combination identified by calculating synergy score is the miRNA pair that is composed of miR-21 and miR-1, a cardiac-enriched miRNA. There are increasing lines of evidence suggesting that miR-1 might play vital roles in MI, providing the functional links between miR-1 and MI [36]–[38]. A large body of evidence has demonstrated that miR-1 is also involved in cardiac hypertrophy, a leading cause of HF, and overexpression of miR-1 inhibits hypertrophic growth of cardiomyocytes [39]. In the present study, we comprehensively investigated and experimentally validated their potential synergy on myocardial apoptosis, hypertrophy and fibrosis (Figure 4 and 5). Besides significant antiapoptotic and antihypertrophic effects, co-transfection of miR-1 and miR-21 also drastically alleviated oxidative stress caused by H_2_O_2_ but remarkably exacerbated myocardial fibrosis. While our finding strongly supports our speculation of their synergistic potential by using synergy score, it prompts that comprehensive consideration should be cautiously taken about the possible side-effects due to the unwanted but enhanced off-target effects in the future development of utilizing miRNAs for the treatment of cardiovascular disease.

While false positive PPIs and wrong miRNA-target interactions are inevitable, our results open up an opportunity that potential miRNA synergy can be easily and effectively disclosed by applying our method of integrating the vast amounts of protein interactomics and miRNA-target interaction data into a single parameter synergy score. Furthermore, our study provides insights into the nature and scale of synergistic gene regulation mediated by miRNAs and has implications in the biological roles of miRNAs in synergy and miRNA-based therapeutics.

## Materials and Methods

### MiRNA-target Interaction and Protein Interactomics Data

The databases miRSel [6] and ExprTargetDB [16] were jointly applied for obtaining reliable miRNA-gene interaction data as described before [7]. After careful analysis of the official symbols of the target genes in the Homo Sapiens approved nomenclature [19], only 99 out of >2000 mature miRNAs encoded in the human genome were recruited for our experimental validation, which were predicted to regulate expression of a minimum number of 50 protein-encoding genes. These miRNAs are listed in Table S2. There were totally 4851 (99×98/2) miRNA pairs in random.

The bioinformatics software Cytoscape 2.8.2 [17] and its plug-in BisoGenet [18] were used for retrieving the experimentally validated global protein interactomics data from multiple public PPI databases as described before [7]. A global protein interaction network was established for the approved gene symbols [19]. However, before further applications the network needed some necessary pruning. Self-loops, isolated nodes and small network components were all removed from the network. Next, another plug-in NetworkAnalyzer assigned nodes representing proteins of the network topology properties [40]. Among all of the calculated topological parameters, two were selected in this study, degree and betweenness centrality. The topological parameter degree counted the number of edges linked to a given node. A high degree value means that the node may represent a hub protein which performs important roles in cellular function [34]. Here, we followed the classification scheme used by Lu *et al*. [41]. If the degree value of a node was ≥20, then it was defined as a network hub. Different from degree, betweenness centrality reflects how extensive control of a given node exerts over the interactions of other nodes in the network [35]. Taken together, it can be expected that hubs of high betweenness centrality values might perform multiple important functions in the network [22]. A threshold value of betweenness centrality at 0.001 was set for the global protein interaction network that was especially built here. Of approximately 11% of the nodes in the network, their betweenness centrality values were higher than 0.001. And only about 7% of the network nodes were assigned with both high degree and betweenness centrality values.

To explore miRNA synergy in restricted conditions, a set of genes with common feature must be available for specific physiology or pathology. In the present study, the database HPRD of Release 9 [15] was used to obtain the protein expression profile of human heart for investigating cardiac miRNA synergy. As described before [7], this protein list encompasses both the proteins that have been experimentally validated to be expressed in human heart and those ubiquitously expressed in the human body. In accordance with the above procedure, we also obtained the protein expression profiles of the other four main human tissues including brain, kidney, liver and lung. In addition, the Gene Prospector online tool was applied to search the literature for obtaining the gene set of apoptosis-related and fibrosis-related genes and the gene sets of MI and HF [14]. Especially, for comprehensively determining the MI-associated genes, the following search keywords were used: myocardial infarction, myocardial ischemia, ischemic heart disease, and coronary heart disease.

### Gene Ontology (GO) Analysis

To determine whether miRNAs could significantly co-regulate the same biological process, we performed GO analysis by applying the online DAVID functional annotation tool [42]. Firstly, the official symbols of all target genes of the 99 miRNAs included in our study were converted into their corresponding Entrez gene IDs. The Entrez gene IDs were then submitted online for indicating the significantly enriched GO biological process that each miRNA might be implicated in as only the category ′GOTERM_BP_5′ was selected. In this study, the GO terms were only considered significantly enriched unless Benjamini-adjusted *p*-value was <0.05 [43]. Finally, we calculated the number of the co-regulated GO terms of every miRNA pairs. According to the number of co-regulated GO terms, the 4851 miRNA pairs were divided into two groups, high co-regulation group (GO term, n >10) and low co-regulation group (GO term, n ≤10).

### Target Similarity Score, Protein Interaction Score and Synergy Score

Except that synergistic actions of miRNAs could happen through targeting common genes [8], functional association between target gene products might be another important contributory factor to synergistic action as revealed in the work of Li *et al.* [44]. They developed a network biology approach to investigate synergistic effects of drug combinations. Hence, in our study both of the above two factors were fully considered for potential miRNA synergy. We supposed that miRNAs could act synergistically due to both targeting common genes and different genes which products were functionally interacted. We defined two quantitative parameters target similarity score (TSS) and protein interaction score (PIS) to respectively determine the above two factors. The following formula is used to calculate TSS for any given miRNA pair miRNA *i* and miRNA *j*:
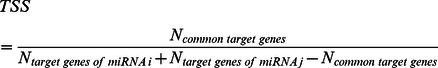



In order to more clearly illustrate the calculation process of PIS, a graphical presentation is provided. As shown in Figure 1, PIS was defined as the sum of proteins divided by the sum of PPIs between protein clusters. The value of PIS could reflect the interaction extent of proteins encoded by the target genes that are respectively regulated by any two miRNAs *i* and *j*. Synergy score is an integrated parameter of TSS and PIS:
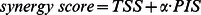



The coefficient α was used as a correction factor for the contribution of PIS to synergy score. GraphPad Prism 5 was applied to generate the ROC curve of synergy score in distinguishing between the high and low co-regulation groups of miRNA pairs. Furthermore, the method robustness of synergy score was examined against miRNA-target interactions and PPIs by applying the miRNA target gene prediction database miRecords [20] and the Human Signaling Network dataset that is updated just recently [21], respectively. Notably, reliable target genes of a given miRNA were accepted only when miRNA-target interactions were simultaneously predicted by at least five target prediction programs in miRecords.

### Cell Culture and Transfection

Neonatal rat ventricular cardiomyocytes (CMs) and cardiac fibroblasts (CFs) were isolated and cultured from 1 to 2-day-old Sprague-Dawley rats, in which use of animals complied with the Guide for the Care and Use of Laboratory Animals published by the US National Institutes of Health (NIH Publication, No. 85–23, revised 1996) and pre-approved by the Experimental Animal Ethic Committee of the Harbin Medical University, China (Animal Experimental Ethical Inspection Protocol, No. 2009104). Briefly, hearts were quickly minced and digested with 0.25% trypsin. The cell suspensions were centrifuged at 2000 rpm for 180 s, then cells were incubated for 2 h in the medium consisted of Dulbecco's Modified Eagle Medium (DMEM), 10% fetal bovine serum, 100 U/ml penicillin and 100 U/ml streptomycin. Neonatal rat CMs were collected and plated in DMEM for another 48 h and CFs passaged by trypsin and used for studies at the 2nd to 4th passage.

CMs and CFs were transfected with miR-1, miR-21, negative control (NC) siRNAs, or miR-1 and miR-21 together using X-treme GENE siRNA transfection reagent (Roche, Switzerland). Transfection concentrations were 50 nM for miR-1, miR-21 and NC siRNAs. Twelve hours after transfection CMs were treated with 10 µM of isoproterenol for 48 h and incubated with 50 µM of H_2_O_2_ for 2 h, respectively. Meanwhile, CFs were exposed to 100 nM of angiotensin II for 48 h. The processed CMs and CFs were then used for miRNA content determination and further functional experiments. Furthermore, the transfection concentration ratios of miR-1 and miR-21 in the concentration range from 10 to 50 nM were assayed to optimize synergistic effect against myocardial apoptosis on H_2_O_2_-treated CMs. The synergistic effect of the randomly matched pairs of miR-20a, miR-21, miR-146a and miR-222 were then investigated on H_2_O_2_-induced cardiomyocytes apoptosis in CMs. Transfection concentrations were 40 nM for each miRNAs and NC siRNAs.

### Acridine Orange/ethidium Bromide (AO/EB ) Staining and MTT Assay

To detect the impact of miRNAs on cell apoptosis, the cultured CMs were washed with PBS and then incubated with 100 µg/mL of AO and EB (Sigma Aldrich, USA) for 5 min. Normal and apoptotic cells were observed under a fluorescence microscope equipped with a CCD digital camera (Nikon Corporation, Japan). MTT assay was conducted to determine the viability of CMs. Briefly, the cultured CMs were plated in 96-well plate. After miRNA transfection and H_2_O_2_ treatment, cells were incubated with 10 µL MTT of 0.5 mg/ml at 37°C for 4 h. The purple formazan crystal was dissolved with 100 µL of dimethyl sulfoxide (DMSO) and added to the cells. The absorbance was measured by spectrophotometer (Tecan Group Ltd., Switzerland) at 570 nm.

### Measurement of SOD Content in CMs and Collagen Content in CFs

The activity of SOD in CMs was measured using the SOD Detection kit (Nanjing Jiancheng Bioengineering Institute, China). Following the manufacturer’s instruction, the results were determined at 550 nm and are expressed as unit per mgport. Collagen content in CFs was assayed with the Sircol™ Collagen assay kit (Biocolor, Ireland) according to the manufacturer’s instruction. Briefly, in a tube for protecting from light CFs were lysed by 100 µL of lysate and then 1 ml of Sircol Dye reagent was added. After cell suspension had been shaken for 40 min, the lysates were cleared by centrifugation at 13500 rpm for 30 min at 4°C. The precipitation was dissolved with proper Alkali reagent and measured at 540 nm in a 96-well plate. Detection values were converted to collagen content that was normalized by the protein concentration of each group.

### Quantitative Reverse Transcription–polymerase Chain Reaction

RNA samples were extracted from CMs and CFs with Trizol reagent (Invitrogen, USA). According to the manufacturer’s protocols, High-Capacity cDNA Reverse Transcription Kit (Applied Biosystems, USA) was used for cDNA synthesis from a total of 0.5 µg RNA. The mRNA levels were quantified with SYBR Green PCR Master Mix Kit and performed on ABI 7500 fast Real Time PCR system (Applied Biosystems, USA). U6 and GADPH were used for template normalization. For sequences details, please see supplemental material (Table S11).

### Statistics for Functional Experiments

All data are expressed as means ± SEM. Statistical analysis was performed using one-way ANOVA followed by Bonferroni’s test. Differences were considered as statistically significant when *p*<0.05.

## Supporting Information

Figure S1 **PIS evaluation of functional protein association level.** Among the 4851 random miRNA-miRNA combinations, the miRNA pair miR-23b:miR-367 was assigned with the minimum PIS score showing sparse functional association between target gene products of miR-23b and miR-367. In comparison, the high PIS score of the miRNA pair miR-21:miR-145 positively reflected dense functional association between proteins that are encoded by their target genes. However, such close functional protein association was not expected for another two miRNA pairs miR-23b:miR-21 and miR-367:miR-145 according to the PIS calculation result. Red, green and yellow nodes represent proteins that are encoded by gene targeted by single miRNAs and both of two, respectively.
(TIF)

Figure S2 **Indicative effect of TSS (A), PIS (B) and synergy score (C) for the number of miRNA co-regulated GO-term processes.**
(TIF)

Figure S3 **Comparison of synergy score distributions of miRNA pairs.**
(TIF)

Figure S4 **Optimization of synergy score.** As the correction factor α of PIS is set at 1.80 (red dot), synergy score possesses the optimal performance in distinguishing between the high and low co-regulation groups of miRNA pairs.
(TIF)

Figure S5 **Dependences of synergy score's performance and correlation factor α on the cutoff of the number of co-regulated GO terms.** A. Box-and-whisker plot of the cutoff and AUC (α = 1.8); B. Box-and-whisker plot of the cutoff and the optimized α. No outliers were found as Whiskers was set 5–95 percentile. The cutoffs of the number of co-regulated GO terms were from 0 to 20.
(TIF)

Figure S6 **Result of the random assignation test (n = 10).** The sum of the validated and predicted target genes of miR-21 was 288 in human genome (see Table S1). In the present test, a total of 288 target genes were randomly selected to undergo the GO analysis. Ten times of GO analyses were performed at each key target gene percentage level.
(TIF)

Figure S7 **Results of miRNA synergy scores in human heart.** A. Semilog line correlation between the number of miRNA target genes and the mean miRNA synergy score (correlation coefficient R = 0.500); B. Semilog line correlation between the number of key miRNA target genes and the mean miRNA synergy score (correlation coefficient R = 0.864). MiR-21 is highlighted as red dot.
(TIF)

Figure S8 **Comparison of genome-wide and cardiac-specific synergistic miRNA pairs.**
(TIF)

Figure S9 **Validation of miR-1 (A) and miR-21 (B) transfection in neonatal rat ventricular cardiomyocytes and cardiac fibroblasts.** NC: negative control; ns: not significant; **p<0.01; n = 5.
(TIF)

Figure S10 **Involvement of genes in MI, HF, and apoptosis.** A. 365 MI-associated genes were also related with apoptosis. B. 517 HF-associated genes were also related with apoptosis.
(TIF)

Table S1 **Results of synergy score calculation in whole human genome.**
(XLSX)

Table S2 **The miRNAs analyzed in the present study.**
(XLSX)

Table S3 **Results of synergy score calculation in whole human genome (miRecords for predicted target gene data).**
(XLSX)

Table S4 **Result of method robustness against miRNA-gene interactions.**
(DOCX)

Table S5 **Result of method robustness against PPI networks.**
(DOCX)

Table S6 **Results of synergy score calculation in human heart.**
(XLSX)

Table S7 **Results of synergy score calculation in apoptosis.**
(XLSX)

Table S8 **The sums of miRNA target genes and miRNA-interactions in restricted conditions.**
(DOCX)

Table S9 **Results of synergy score calculation in MI.**
(XLSX)

Table S10 **Results of synergy score calculation in HF.**
(XLSX)

Table S11 **Detailed sequences of the essential primers used in the present study.**
(DOCX)

Table S12 **Results of synergy score calculation in human brain.**
(XLSX)

Table S13 **Results of synergy score calculation in human kidney.**
(XLSX)

Table S14 **Results of synergy score calculation in human liver.**
(XLSX)

Table S15 **Results of synergy score calculation in human lung.**
(XLSX)

Table S16 **Databases, online tools and software used in the present study.**
(DOCX)
