# Supplementary figures and images for: Dissection of Protein Interactomics Highlights MicroRNA Synergy
Source: PLoS One. 2013 May 14;8(5):e63342. doi: 10.1371/journal.pone.0063342 (PMC3653946; doi:10.1371/journal.pone.0063342)

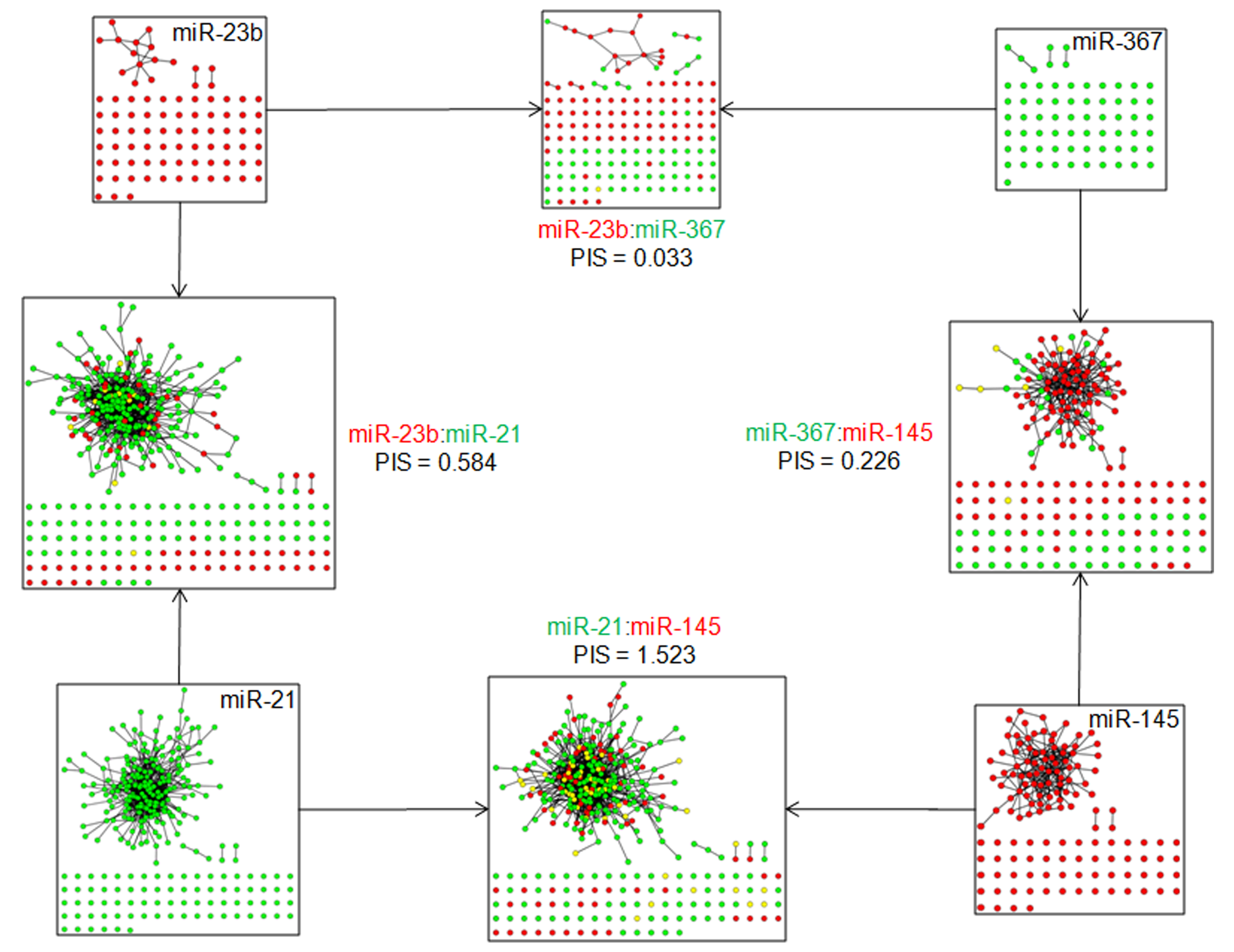

Supplement: Figure S1 — PIS evaluation of functional protein association level. Among the 4851 random miRNA-miRNA combinations, the miRNA pair miR-23b:miR-367 was assigned with the minimum PIS score showing sparse functional association between target gene products of miR-23b and miR-367. In comparison, the high PIS score of the miRNA pair miR-21:miR-145 positively reflected dense functional association between proteins that are encoded by their target genes. However, such close functional protein association was not expected for another two miRNA pairs miR-23b:miR-21 and miR-367:miR-145 according to the PIS calculation result. Red, green and yellow nodes represent proteins that are encoded by gene targeted by single miRNAs and both of two, respectively. (TIF) [file pone.0063342.s001.tif]

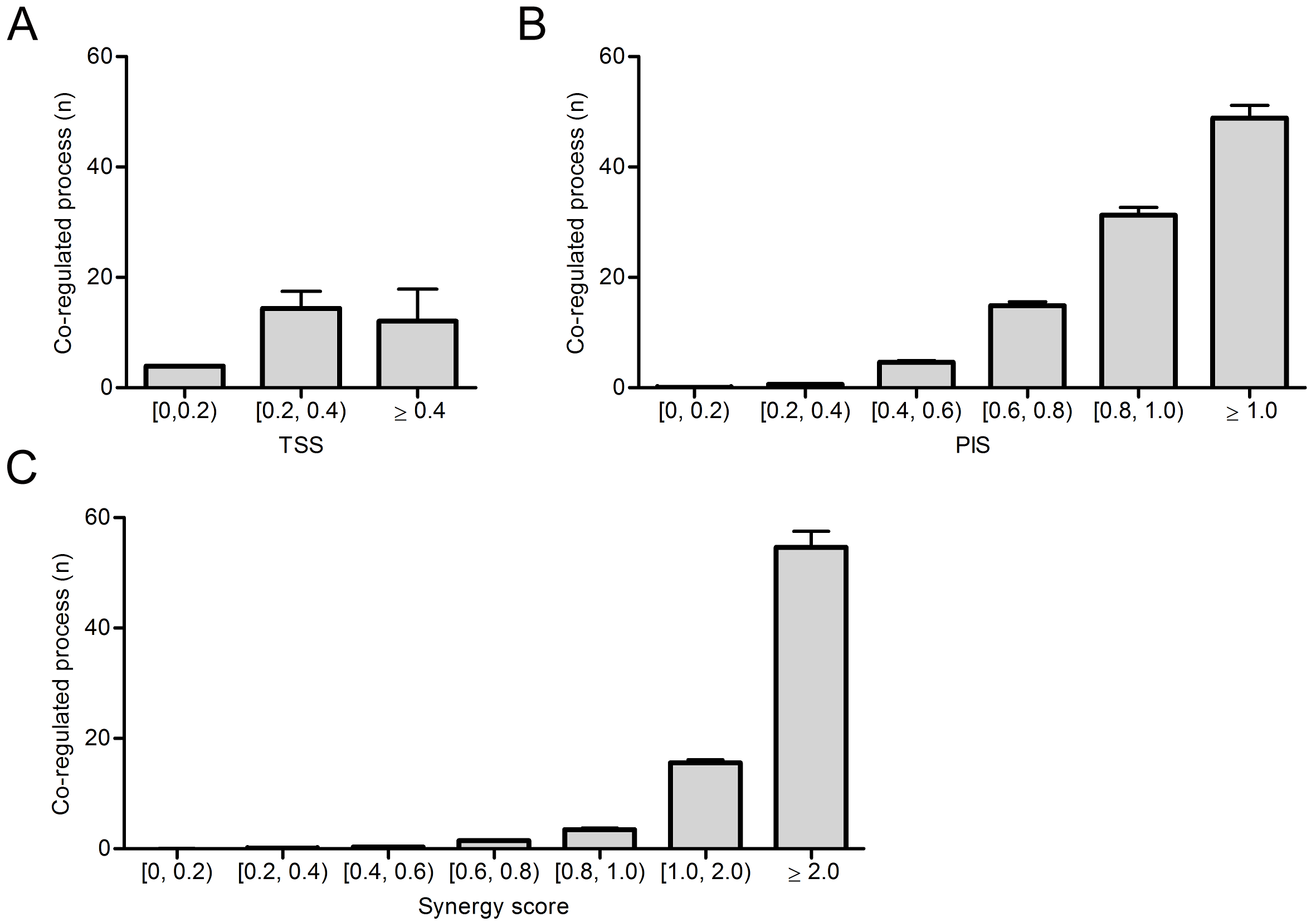

Supplement: Figure S2 — Indicative effect of TSS (A), PIS (B) and synergy score (C) for the number of miRNA co-regulated GO-term processes. (TIF) [file pone.0063342.s002.tif]

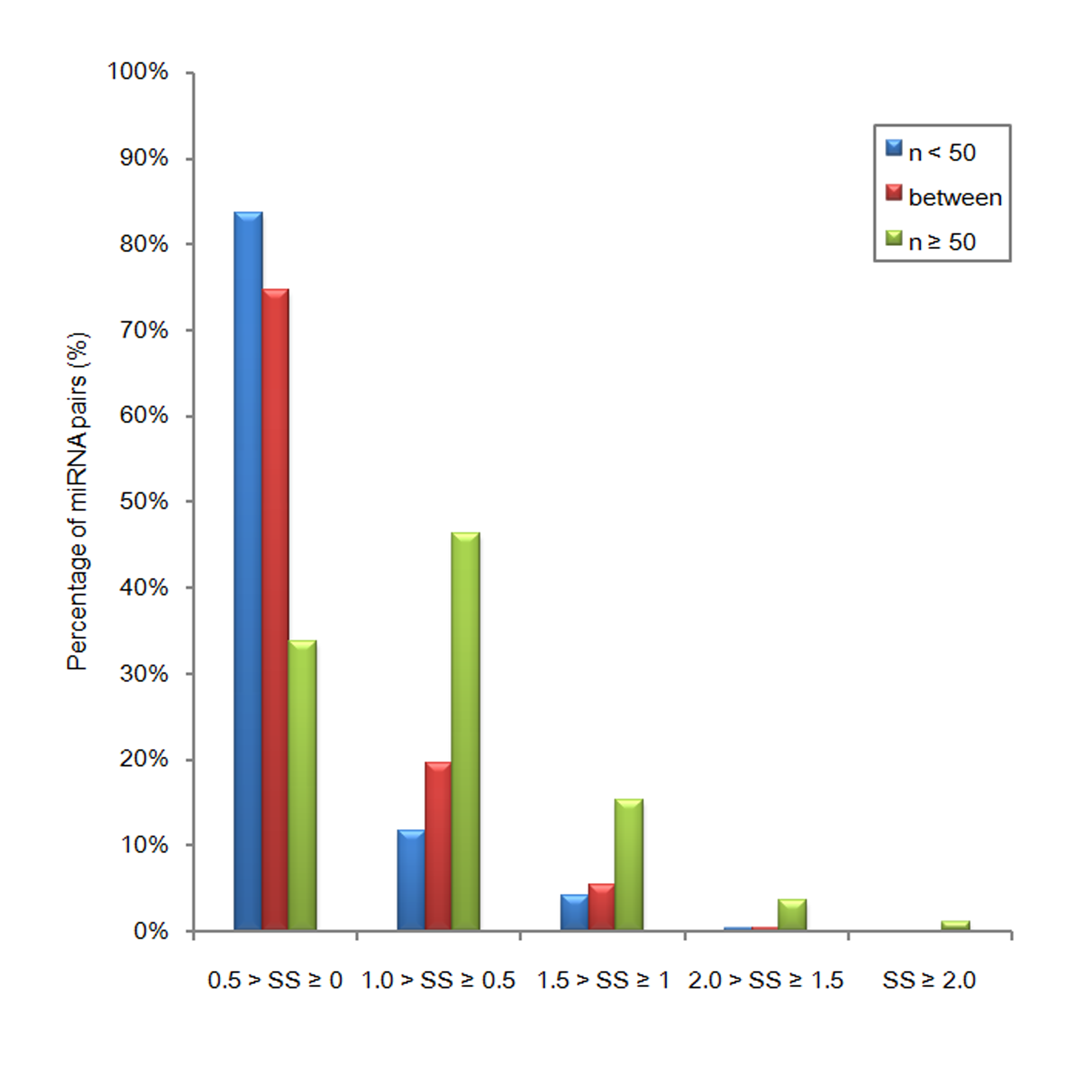

Supplement: Figure S3 — Comparison of synergy score distributions of miRNA pairs. (TIF) [file pone.0063342.s003.tif]

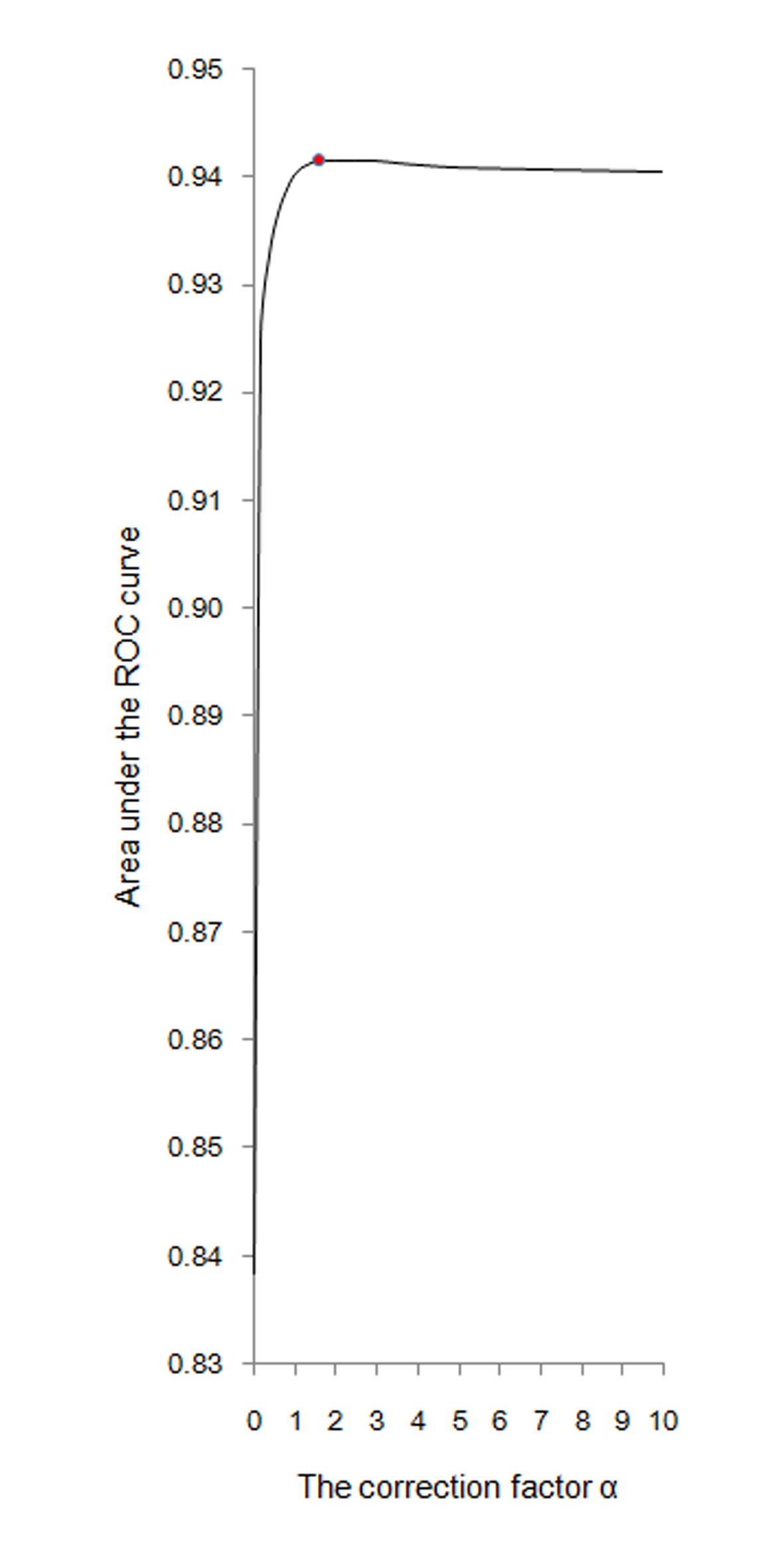

Supplement: Figure S4 — Optimization of synergy score. As the correction factor α of PIS is set at 1.80 (red dot), synergy score possesses the optimal performance in distinguishing between the high and low co-regulation groups of miRNA pairs. (TIF) [file pone.0063342.s004.tif]

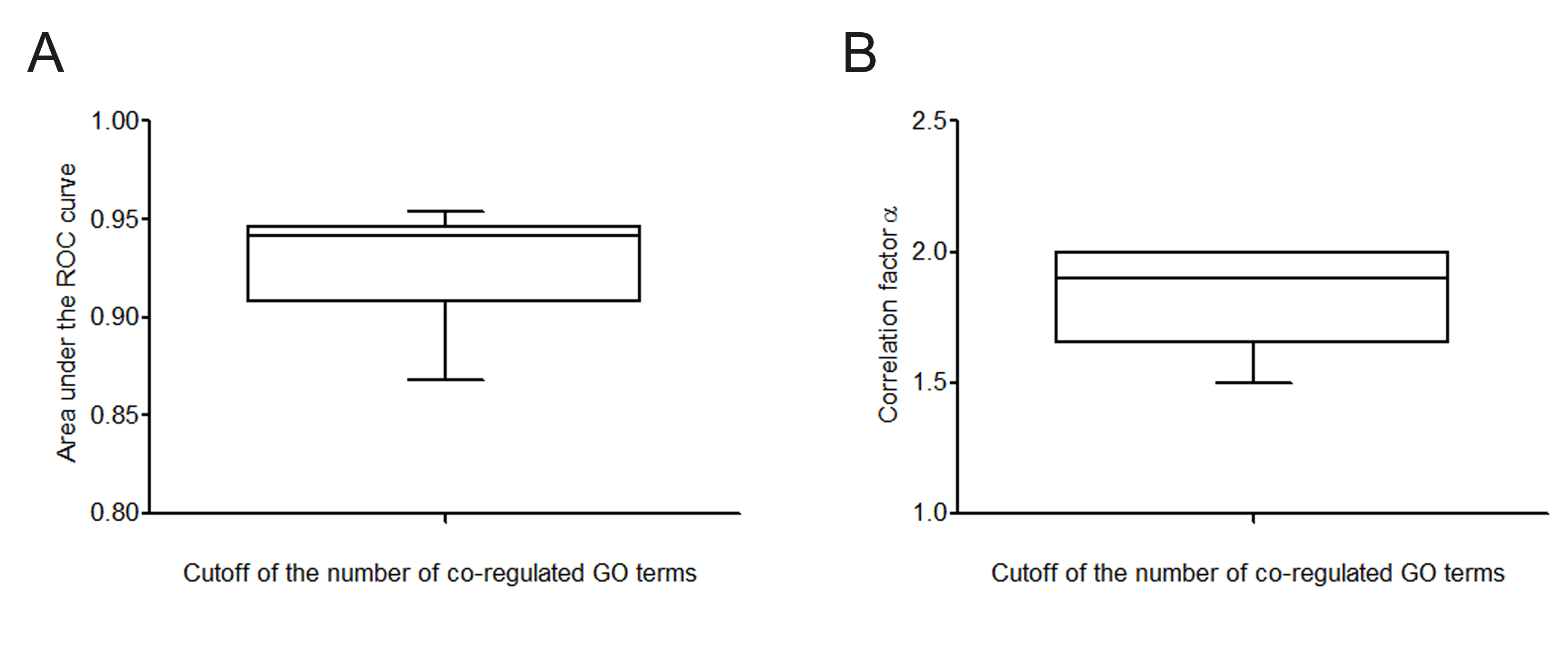

Supplement: Figure S5 — Dependences of synergy score's performance and correlation factor α on the cutoff of the number of co-regulated GO terms. A. Box-and-whisker plot of the cutoff and AUC (α = 1.8); B. Box-and-whisker plot of the cutoff and the optimized α. No outliers were found as Whiskers was set 5–95 percentile. The cutoffs of the number of co-regulated GO terms were from 0 to 20. (TIF) [file pone.0063342.s005.tif]

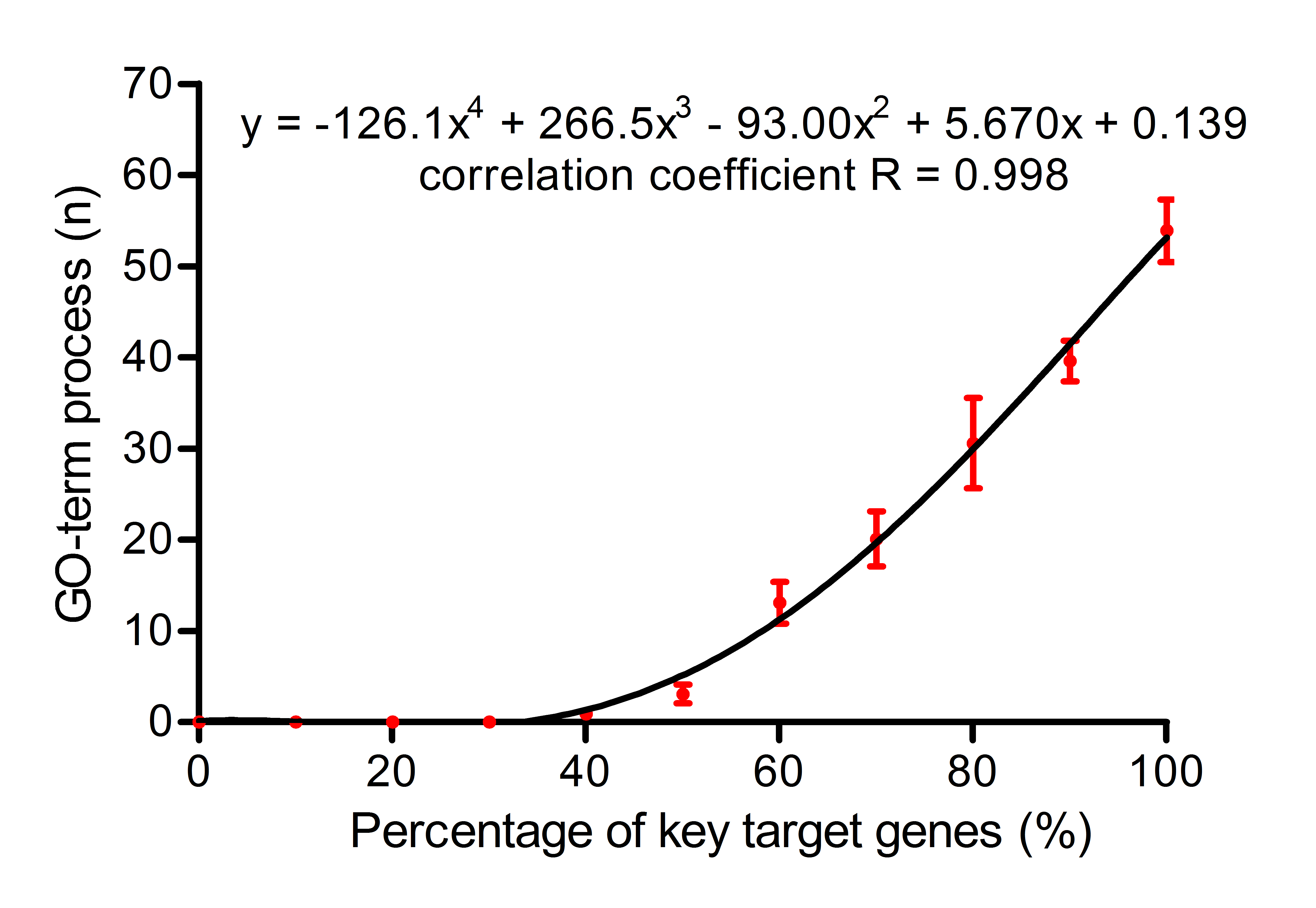

Supplement: Figure S6 — Result of the random assignation test (n = 10). The sum of the validated and predicted target genes of miR-21 was 288 in human genome (see Table S1). In the present test, a total of 288 target genes were randomly selected to undergo the GO analysis. Ten times of GO analyses were performed at each key target gene percentage level. (TIF) [file pone.0063342.s006.tif]

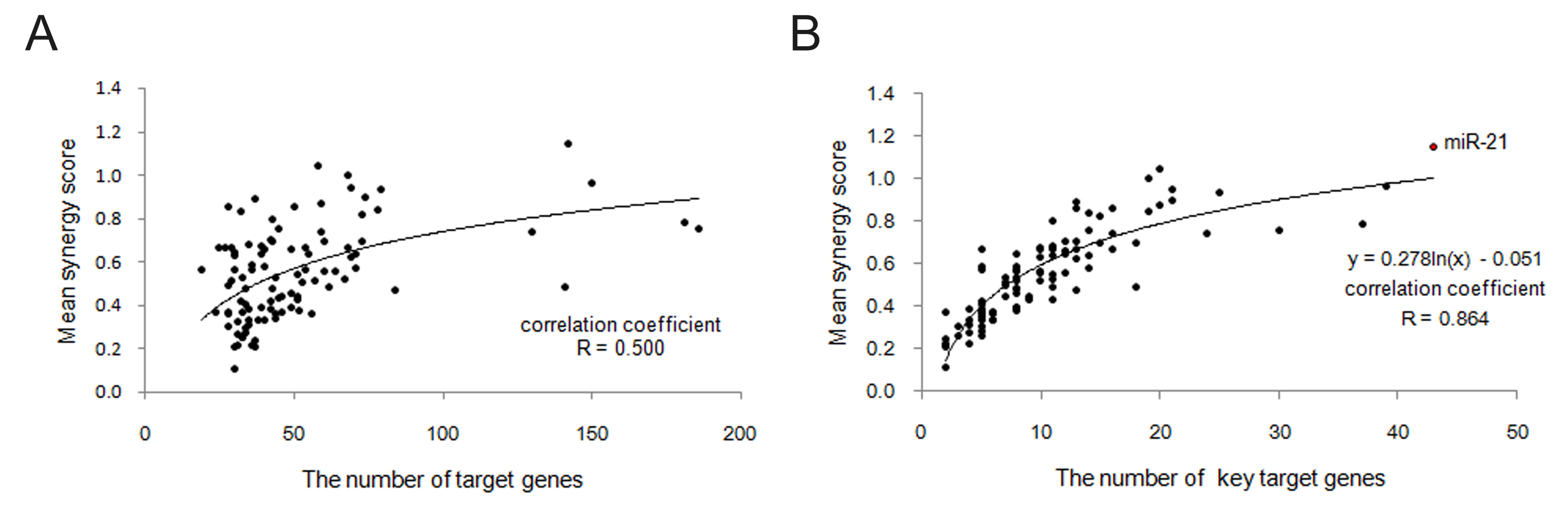

Supplement: Figure S7 — Results of miRNA synergy scores in human heart. A. Semilog line correlation between the number of miRNA target genes and the mean miRNA synergy score (correlation coefficient R = 0.500); B. Semilog line correlation between the number of key miRNA target genes and the mean miRNA synergy score (correlation coefficient R = 0.864). MiR-21 is highlighted as red dot. (TIF) [file pone.0063342.s007.tif]

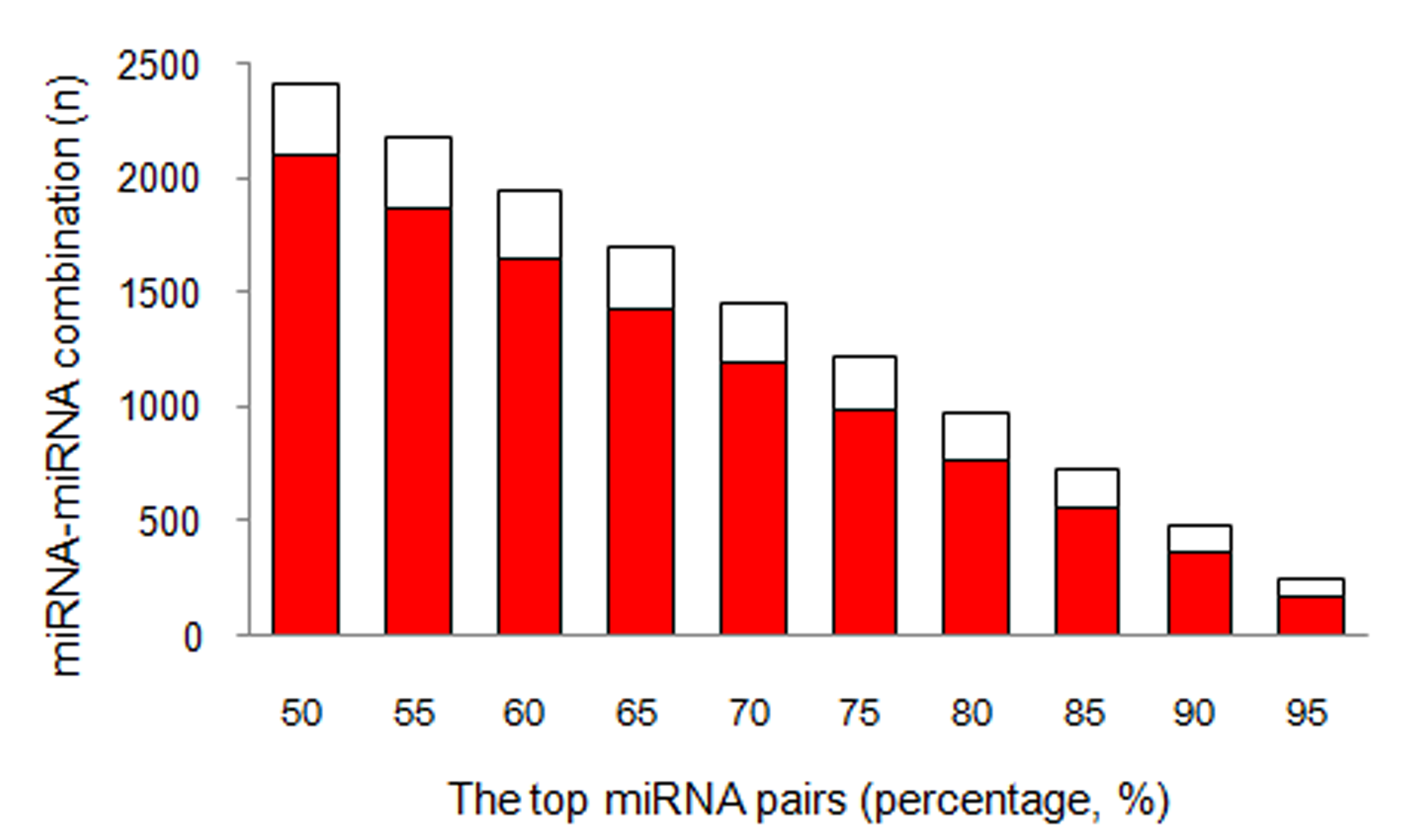

Supplement: Figure S8 — Comparison of genome-wide and cardiac-specific synergistic miRNA pairs. (TIF) [file pone.0063342.s008.tif]

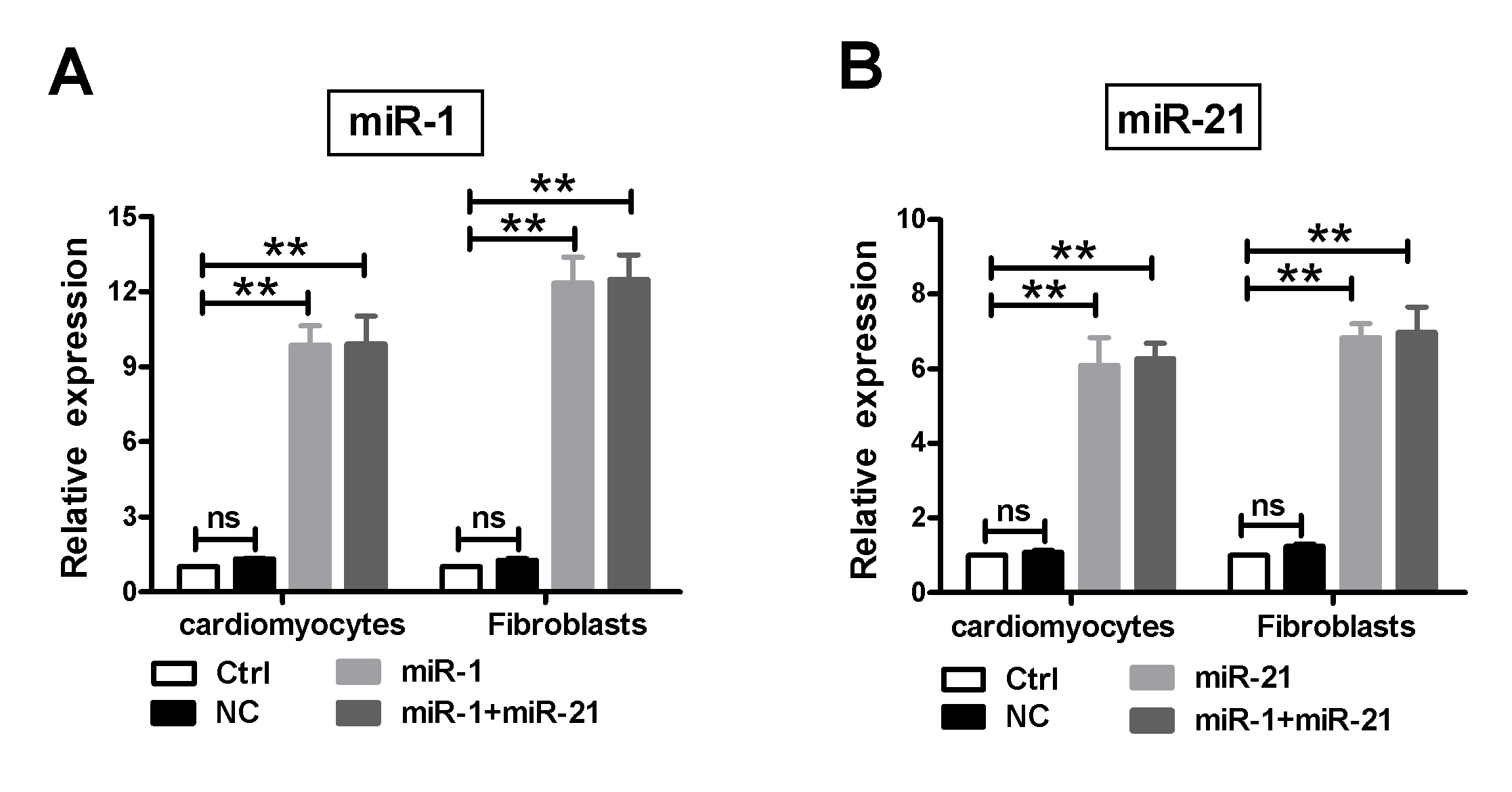

Supplement: Figure S9 — Validation of miR-1 (A) and miR-21 (B) transfection in neonatal rat ventricular cardiomyocytes and cardiac fibroblasts. NC: negative control; ns: not significant; **p<0.01; n = 5. (TIF) [file pone.0063342.s009.tif]

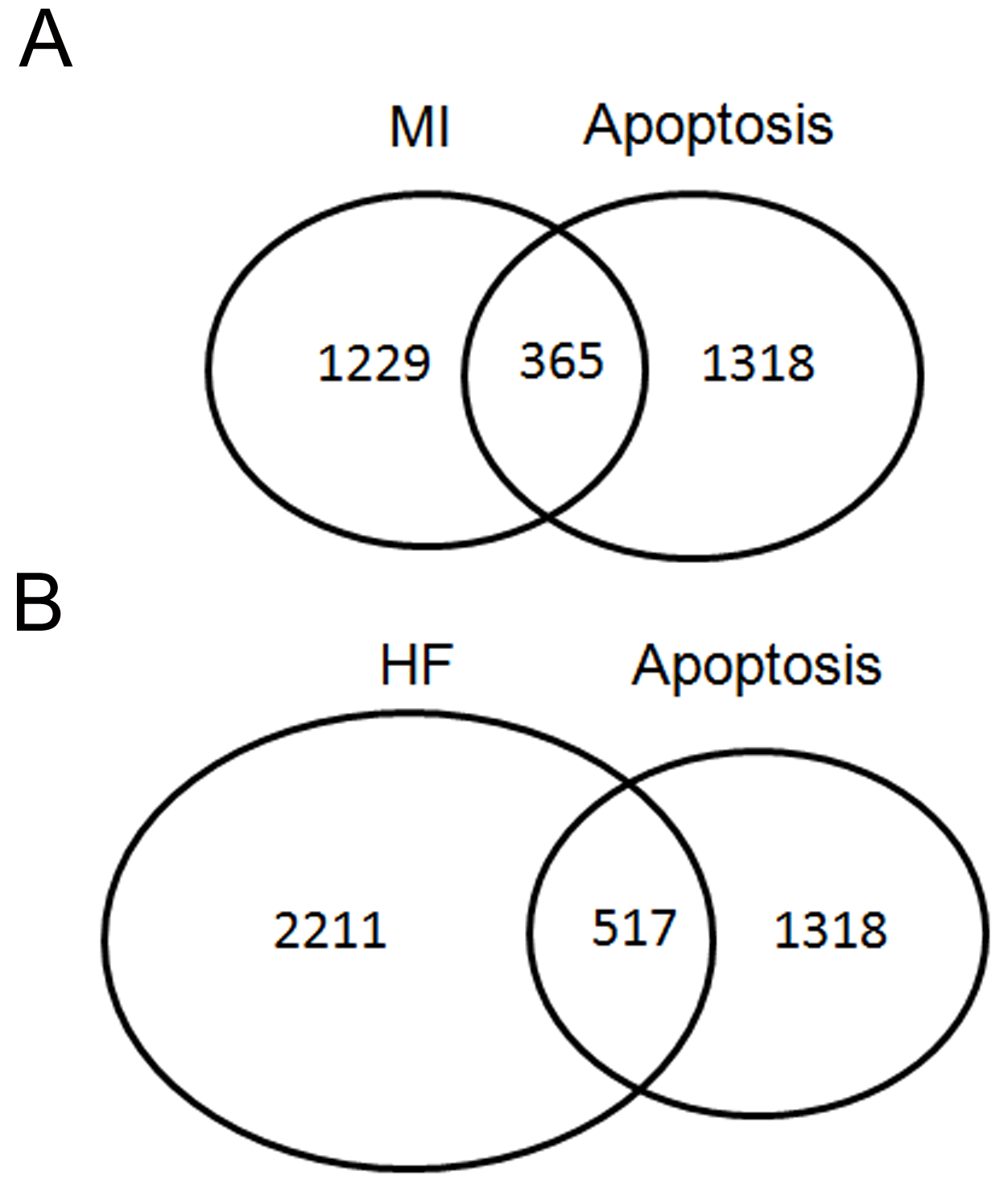

Supplement: Figure S10 — Involvement of genes in MI, HF, and apoptosis. A. 365 MI-associated genes were also related with apoptosis. B. 517 HF-associated genes were also related with apoptosis. (TIF) [file pone.0063342.s010.tif]
