# Supplementary material for: Dissection of Protein Interactomics Highlights MicroRNA Synergy
Source: PLoS One. 2013 May 14;8(5):e63342. doi: 10.1371/journal.pone.0063342 (PMC3653946; doi:10.1371/journal.pone.0063342)
Supplement: Table S4 — Result of method robustness against miRNA-gene interactions. (DOCX) [file pone.0063342.s014.docx]

**Table S4.** Result of method robustness against miRNA-gene interactions.

| miRNA pair | Rank (miRSel+ExprTargetDB) | Rank (miRSel+miRecords) |
| --- | --- | --- |
| miR-21:miR-155 | 1 | 1 |
| miR-21:miR-145 | 2 | 4 |
| miR-1:miR-21 | 3 | 5 |
| miR-21:miR-222 | 4 | 3 |
| miR-16:miR-21 | 5 | 7 |
| miR-21:miR-206 | 6 | 8 |
| miR-21:miR-146a | 7 | 2 |
| miR-21:miR-143 | 8 | 19 |
| miR-21:miR-221 | 9 | 6 |
| miR-21:miR-200b | 10 | 10 |
| miR-21:miR-125b | 11 | 21 |
| miR-15a:miR-21 | 12 | 17 |
| miR-145:miR-155 | 13 | 9 |
| miR-20a:miR-21 | 14 | 31 |
| miR-146a:miR-155 | 15 | 14 |
| miR-1:miR-155 | 16 | 11 |
| miR-155:miR-222 | 17 | 15 |
| miR-155:miR-206 | 18 | 18 |
| miR-146a:miR-222 | 19 | 27 |
| miR-21:miR-223 | 20 | 29 |
