# Supplementary material for: Dissection of Protein Interactomics Highlights MicroRNA Synergy
Source: PLoS One. 2013 May 14;8(5):e63342. doi: 10.1371/journal.pone.0063342 (PMC3653946; doi:10.1371/journal.pone.0063342)
Supplement: Table S8 — The sums of miRNA target genes and miRNA-interactions in restricted conditions. (DOCX) [file pone.0063342.s018.docx]

**Table S8.** The sums of miRNA target genes and miRNA-interactions in restricted conditions.

| restricted condition | sum of genes ( targeted by miRNA, %) | sum of miRNA-target interactions (density) |
| --- | --- | --- |
| whole genome | 19045 (3541, 18.6%) | 10235 (2.89) |
| heart | 7535 (1810, 24.0%) | 5100 (2.82) |
| apoptosis | 1318 (508, 38.5%) | 2396 (4.72) |
| MI | 1229 (418, 34.0%) | 1705 (4.08) |
| HF | 2211 (728, 32.9%) | 3000 (4.12) |

The sums of miRNA target genes and miRNA-interactions in restricted conditions. Density is the ratio between the sum of miRNA-gene interactions and the sum of genes targeted by miRNAs.
