# Supplementary material for: Dissection of Protein Interactomics Highlights MicroRNA Synergy
Source: PLoS One. 2013 May 14;8(5):e63342. doi: 10.1371/journal.pone.0063342 (PMC3653946; doi:10.1371/journal.pone.0063342)
Supplement: Table S11 — Detailed sequences of the essential primers used in the present study. (DOCX) [file pone.0063342.s021.docx]

**Table S11.** Detailed sequences of the essential primers used in the present study.

| Gene | Sequence |
| --- | --- |
| GAPDH | Forward: 5'-AAGAAGGTGGTGAAGCAGGC -3' |
|  | Reverse: 5'-TCCACCACCCAGTTGCTGTA -3' |
| U6 | Forward: 5'-GCTTCGGCACATATACTAAAAT-3' |
|  | Reverse: 5'-CGCTTCACGAATTTGCGTGTCAT-3' |
| β-MHC | Forward: 5'-AACCTGTCCAAGTTCCGCAAGGTG-3' |
|  | Reverse: 5'-GAGCTGGGTAGCACAAGAGCTACT-3' |
| ANP | Forward: 5'-TCAGAGAGATGGAGGTGCT-3'  Reverse: 5'-CCAATCCTGTCAATCCTAC-3' |
| BNP | Forward: 5'-TGATTCTGCTCCTGCTTTTC -3'  Reverse: 5'-GTGGATTGTTCTGGAGACTG -3' |
| rno-miR-1 | Forward: 5'- GGGGTGGAATGTAAAGAAGTG-3' |
|  | Reverse: 5'- CGTGGAGTCGGCAATTGCA -3' |
| rno-miR-21 | Forward: 5'-GGGGTAGCTTATCAGATCG -3' |
|  | Reverse: 5'-TGGAGTCGGCAATTGCACTG -3' |

Detailed sequences of the essential primers used in this study. U6 was used as an internal control of template normalization for miR-1 and miR-21 and GADPH for ANP, BNP and β-MHC. ANP: Atrial natriuretic peptide; BNP: brain natriuretic peptide; β-MHC: beta myosin heavy chain.
