# Supplementary material for: Dissection of Protein Interactomics Highlights MicroRNA Synergy
Source: PLoS One. 2013 May 14;8(5):e63342. doi: 10.1371/journal.pone.0063342 (PMC3653946; doi:10.1371/journal.pone.0063342)
Supplement: Table S16 — Databases, online tools and software used in the present study. (DOCX) [file pone.0063342.s026.docx]

**Table S16.** Databases, online tools and software used in the present study.

| Resource name | Website link |
| --- | --- |
| ExperTargetDB^a^ | http://www.scandb.org/apps/microrna/index.html |
| HGNC^a^ | http://www.genenames.org/ |
| HPRD^a^ | http://www.hprd.org/ |
| Human Signaling Network^a^ | http://www.bri.nrc.ca/wang/ |
| miRecords^a^ | http://mirecords.umn.edu/miRecords/ |
| miRSel^a^ | http://services.bio.ifi.lmu.de/mirsel/ |
| Gene Prospector online tool^b^ | http://www.hugenavigator.net/HuGENavigator/geneProspectorStartPage.do |
| DAVID functional annotation tool^b^ | http://david.abcc.ncifcrf.gov/ |
| Cytoscape^c^ | http://www.cytoscape.org/ |
| BisoGenet^c^ | http://bio.cigb.edu.cu/bisogenet-cytoscape/ |
| NetworkAnalyzer^c^ | http://med.bioinf.mpi-inf.mpg.de/netanalyzer/ |

Databases, online tools and software used for synergy score calculation. a: database; b: online tool; c: software. BisoGenet and NetworkAnalyzer are the plugins of Cytoscape. HGNC: [hugo gene nomenclature committee](http://www.baidu.com/link?url=pG9JGJqjJ4zBBpC8yDF8xDh8vibi1ltfEWoCbI1C2tuhL9_); HPRD: Human Protein Reference Database.
